# Public Knowledge and Adherence to Hand Hygienic Guidelines for the Prevention of SARS-CoV-2 Transmission: A Cross-sectional Survey from Pakistan

**DOI:** 10.1017/dmp.2021.92

**Published:** 2021-03-25

**Authors:** Muhammad Osama Yaseen, Arifa Saif, Tahir Mehmood Khan, Misha Yaseen

**Affiliations:** 1 Institute of Pharmaceutical Sciences, University of Veterinary and Animal Sciences, Lahore, Pakistan; 2 School of Pharmacy, Monash University, Bandar Sunway, Selangor, Malaysia; 3 CMH Kharian Medical College, Kharian, Pakistan

**Keywords:** coronavirus, COVID-19, hand hygiene, public health/risk factors

## Abstract

**Objective:**

Good hand hygienic practices are considered an important factor to curb the transmission and emergence of SARS-CoV -2. Various studies, conducted previously during the outbreaks of SARS-CoV and MERS-CoV, have ascertained the effectiveness of adopting good hand hygienic practices to curb the emergence of these viruses. This study aims to explore public hand hygienic practices during the peak pandemic period.

**Method:**

A descriptive cross-sectional study was conducted among the general population of Pakistan to investigate the knowledge and perception about hand hygiene, self-reported hand hygiene practices, adherence to hand hygienic guidelines, and barriers to optimal hand hygiene. *Kruskal-Wallis test, Mann-Whitney U test, and Regression model* were used for statistical analysis.

**Results:**

There was a significant difference in area-based knowledge (*P* = 0.026), beliefs (*P* = 0.027), and practices (*P* = 0.002) regarding hand hygiene. The results of regression analysis revealed that people in urban areas were more likely to have better knowledge (β = 0.108, CI = 0.076 − 0.05, *P* = 0.008) and better adherence (β = 0.115, CI = 0.514 − 2.68, *P* = 0.004) to hand hygienic practices.

**Conclusion:**

Advertisements on television and other electronic media with appealing slogans could be effective in making people more compliant to optimal hand hygienic practices.

## Introduction

Coronavirus class of pathogens consists of different types of zoonotic agents, some of which had created very serious problems for humans in the past; however, the recent outbreak of severe acute respiratory syndrome-coronavirus-2 (SARS-CoV-2) surpassed all previous coronavirus outbreaks in terms of the rate of transmission and fatality.^1^ SARS-CoV-2 is responsible for a potentially fatal disease, called coronavirus disease (Covid-19), that is surging throughout the globe and challenging the healthcare systems of the world without any discrimination.^2^ Covid-19 first originated in November 2019 in Wuhan city of China as pneumonia of unknown cause, reported to WHO country office in December 2019, and later declared by WHO as worldwide public health emergency in March 2020.^3^

By the end of February 2020, coronavirus had made its entry into Pakistan through individuals with a travel history from countries that were badly affected by the novel coronavirus outbreak.^4^ Local transmission of the virus started due to lack of national level campaigns regarding personal hygiene, frequent disinfection, use of face masks, and keeping social distance. When the pandemic had already shown its colors in Pakistan, the National Institute of Health in Pakistan then devised protocols for prevention of further transmission of coronavirus and launched campaigns for public awareness on the use of personal protective equipment and frequent hand sanitization.^5^

Hands are the most widely recognized vehicle for the transmission of pathogens from an infected person to a healthy individual.^6^ According to WHO, hand hygiene plays a vital role in preventing various infectious diseases.^7^ Studies have demonstrated that millions of annual deaths can be avoided by following good hygiene practices.^8^ The use of antiseptics for hand cleansing after contact with a patient reduces the risk of disease transmission.^9^ Various guidelines have been developed on hand hygiene that recommend the use of alcohol-based hand sanitizer (minimum 60%) or mechanical hand washing for the removal of pathogens, thus preventing their transmission.^10^ Several studies conducted previously during the outbreaks of SARS-CoV and MERS-CoV, have ascertained the effectiveness of adopting good hand hygienic practices along with certain other measures to curb the emergence of these viruses.^11^ A meta-analysis has shown that the rate of upper respiratory tract infections can be reduced up to 21% only by adhering to hand hygiene guidelines.^12^ Evidence suggests that despite having knowledge about hand hygiene practices, adherence to these practices is still challenging.^13^

Addressing the situation in Pakistan, there are hardly any studies that explore the hand hygiene practices among the general public. Therefore, the aim of this study is to explore public knowledge and perceived beliefs regarding hand hygiene which will help in assessing their association with self-reported hand hygienic practices. Moreover, this study will also assess public adherence to hygienic guidelines and various factors that serve as barrier to compliance with optimal hand hygienic practices.

## Methodology

A descriptive cross-sectional online study was conducted among the general population of Pakistan to probe the knowledge and perception about hand hygiene, self-reported hand hygiene practices, adherence to hand hygienic guidelines, and barriers to optimal hand hygiene. Ethical approval for this survey was provided by University of Veterinary and Animal Sciences Lahore, Pakistan. The purpose of this survey was clearly explained in the description before asking the consent of the participants. Information about the participant’s name, national ID card number, mobile number, and e-mail address was avoided to earn the trust of the participant.

### Study design

The study tool was designed to assess different parameters related to hand hygiene during the peak pandemic period from May to June, 2020. The preliminary questionnaire was devised based on extensive literature review, research experience, and a combination of the Human Belief Model and the Theory of Planned Behavior.^14-16^ Items included in the study completely explain the range of beliefs, practices, knowledge, adherence, and barriers related to hand hygiene without overburdening the participants with a lengthy study. Content and face validity measures were adapted to finalize a 31-item questionnaire excluding demographics. The scale was tested by conducting pilot study on 50 individuals from the target population. These individuals could give suggestions on readability, understandability and appropriateness of the items. Items were slightly modified based on wording and format given by the suggestions of individuals. The responses were then coded and subjected to reliability and validity testing using Statistical Package for Social Sciences (SPSS) version 21® (IBM Corporation, Armonk, NY).

#### Reliability

Internal consistency, inter-item correlation, and item-total correlation were applied to assess the reliability of the items. The acceptable Cronbach’s alpha coefficient was considered greater than 0.7 for internal consistency of each sub-scale. Homogeneity of the scale was examined using inter-item correlation and item-total correlation. Acceptable limit for coefficient of inter-item correlation and item-total correlation was considered 0.3 − 0.7 and greater than 0.3 respectively. A coefficient greater than 0.7 stipulates redundancy, while coefficients less than a value of 0.3 mean that items have no contribution to the scale.

#### Validity

Exploratory factor analysis (EFA) was employed to confirm the validity of each sub-scale. Extraction method of principle component analysis, with Varimax rotation was used to perform exploratory factor analysis. Scree plot, total variance explained, and Eigenvalues greater than 1 were the benchmark for factor extraction. The cut-off point for characterizing factor associated variables was greater than 0.4. The study tool was mainly comprised of 6 sections, with first section aiming to explore demographic information while the remaining 5 were aiming to explore knowledge about hand hygiene, beliefs regarding hand hygienic practices, self-reported hand hygienic practices, adherence to hygienic guidelines, and barriers to achieve optimal hand hygiene. The strategy used in each of these sections is discussed below:

#### Knowledge

This sub-scale was made to assess the knowledge of the participants about hand hygiene. There are 7 items in this sub-scale, each with 3 options. Each positive answer will get 1 score while each negative answer will get 0 score. Total score for this sub-scale ranges from 0 to 7. Higher scores indicate more knowledge regarding hand hygiene during the current pandemic and vice versa.

#### Beliefs

This sub-scale was made to assess the potential behavioral, cognitive, and social beliefs of participants regarding hand hygiene. It includes 7 items with Likert scale options ranging from ‘strongly disagree’ (a score of 0) to ‘strongly agree’ (a score of 3) and the total score of this sub-scale is 21. Higher scores indicate positive beliefs towards hand hygiene and vice versa.

#### Self-reported practices

This sub-scale was designed to investigate the self-reported hand hygienic practices of the participants. It includes 8 items with Likert scale options and total score ranging from 0 to 24. Higher scores on this sub-scale indicate good hand hygienic practices and vice versa.

#### Adherence

This sub-scale assesses the participants’ adherence to optimal hand hygienic guidelines. This sub-scale consists of 9 items with options ranging from ‘never’ (score of 0) to ‘always’ (score of 3) and a total score ranging from 0 to 27. Higher score indicates good adherence to guidelines and vice versa.

### Sample size

Raosoft® online calculator (Raosoft Inc., Seattle, WA) was used to calculate the sample size for this study.^17^ The parameters for the calculation of sample size were response distribution, population size, margin of error, and critical value for confidence level. The confidence level was set at 95% while the margin of error was 5%. The recommended size of the sample was 384 to achieve the desired level of confidence, but to give the locals more chance to participate in the study, and to make the findings of the study more precise, this study was conducted on 650 individuals.

### Data analysis

A specific serial number was assigned to every circulated questionnaire to ensure traceability. The entire response was coded and given scores to check the hypothesis using SPSS version 21® (IBM Corporation, Armonk, NY). The entire data was subjected to *normality test* with a cut-off significant value of 0.05. Quantitative analysis of the data was performed using descriptive analysis. For the variables that were not normally distributed on independent factors, Kruskal-Wallis test, and Mann-Whitney U test was used to determine differences among various groups. Regression model was used to assess the association among different variables.

## Results

### Socio-demographic characteristics of the respondents

Around 650 volunteers were approached, 616 participants returned the questionnaire that fulfil the requirements for further interpretations, resulting in a response rate of 94.7%. Of the total 616 participants, 178 (28.9%) were male, 432 (70.1%) were female and 6 (1.0%) respondents preferred not to mention their gender. Of the total participants, 139 (22.6%) were rural inhabitants and 477 (77.4%) were urban residents. Respondents were also categorized based on their education level, and the terms for different education levels were coined according to local education system of Pakistan. Details of socio-demographic characteristics of the respondents are given in Table 1.

**Table 1.** Socio-demographic characteristics of the respondents

Demographics		N (%)
**Age** Mean = 24.137 ± 6.28 [11 – 69 years]	11-20	148 (24.0%)
	21-30	416 (67.5%)
	31-40	33 (5.4%)
	41-50	11 (1.8%)
	>50	8 (1.3%)
**Gender**	Male	178 (28.9%)
	Female	432 (70.1%)
	Prefer not to say	6 (1.0%)
**Area**	Rural	139 (22.6%)
	Urban	477 (77.4%)
**Education**	None	4 (0.6%)
	No certificate but can read/write	3 (0.5%)
	Matriculation	15 (2.4%)
	Intermediate	102 (16.6%)
	University graduate	410 (66.6%)
	Above	82 (13.3%)
**Marital status**	Married	109 (17.7%)
	Single	507 (82.3%)
**Employment**	Employed	187 (30.4%)
	Unemployed	429 (69.6%)

### Instrument reliability and validity

**Figure 1.**
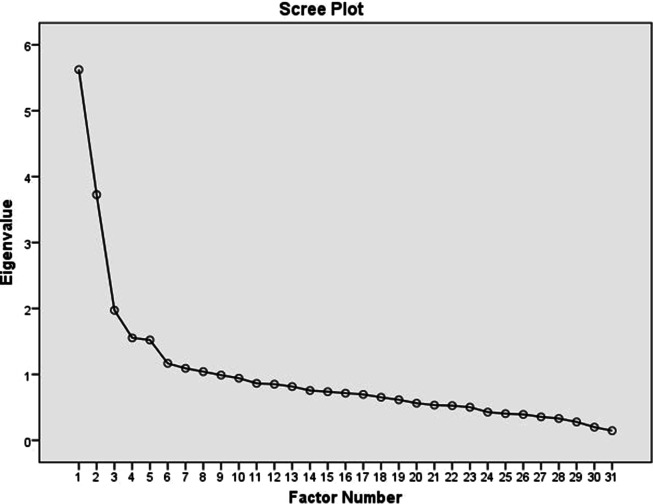
Scree plot authenticating factorability of the scale based on Eigenvalues of more than 1.

Cronbach’s alpha for each of the scales was 0.814, supporting the reliability analysis and indicating good internal consistency of the scale. Internal consistency was calculated for 5 sub-scales, corresponding to 5 factors extracted from exploratory factor analysis. All inter-item correlations were between 0.3 and 0.7, while all item-total correlations were more than 0.3. The 31-item scale was also subjected to principal component analysis. Before analyzing principal component analysis, the sustainability of the scale was assessed using correlation-matrix (> 0.3), Kaiser Meyer-Oklin test (0.84), and Bartlett’s Test of Sphericity (*P* < 0.01), which approved the factorability and recruitment for further factor analysis by principal component analysis. Based on conceptual considerations, scree plot (shown in Figure 1), eigenvalues of greater than 1, and total variance explained, 5 factors were rotated by applying varimax rotation technique. These 5 factors were named knowledge, beliefs, practices, adherence, and barriers as preconceived.

#### Knowledge, beliefs, and practices regarding hand hygiene

Mean scores of the participants are given in Table 2. People with low education tend to have low mean scores with respect to their knowledge and beliefs regarding hand hygiene. Moreover, their hand hygienic practices were unsatisfactory when compared to the people with high educational records. There was a significant difference in area-based knowledge (*P* = 0.026), beliefs (*P* = 0.027), and practices (*P* = 0.002) when the data was subjected to Mann-Whitney U test. Statistically significant difference (*P* = 0.041) was also noticed regarding hand hygienic practices among participants of different educational backgrounds when Kruskal-Wallis test was applied on an abnormally distributed data. Different age groups had difference (*P* = 0.036) in beliefs regarding the importance of optimal hand hygiene. The results of regression analysis revealed that people in urban areas were more likely to have better knowledge (β = 0.108, CI = 0.076 − 0.05, *P* = 0.008) and better adherence (β=0.115, CI =0.514 − 2.68, *P* = 0.004) to hand hygienic practices as shown in Table 3. Moreover, there was a significant association of people’s beliefs (β = 0.157, CI = 0.302 − 0.905, *P* < 0.01) and practices (β = 0.043, CI = 0.105 − 0.353, *P* = 0.003) with their knowledge.

**Table 2.** List of mean scores of respondents and their differences among various groups

Demographics		Knowledge score	p value	Belief score	p value	Practices score	p value	Adherence score	p value
Age	11 - 20 years	4.72 ± 1.171	0.769	16.20 ± 4.255	0.036*	20.32 ± 3.098	0.198	18.84 ± 5.626	0.530
	21 - 30 years	4.74 ± 1.119		16.50 ± 4.366		20.43 ± 3.344		18.56 ± 5.946	
	31 - 40 years	4.91 ± 1.128		17.82 ± 3.980		20.76 ± 2.873		18.97 ± 5.537	
	41 - 50 years	4.64 ± 0.674		17.18 ± 23.16		18.64 ± 3.009		16.91 ± 4.323	
	> 50 years	5.13 ± 1.246		17.63 ± 7.190		19.75 ± 3.012		20.38 ± 5.605	
Gender	Male	4.77 ± 1.051	0.947	16.40 ± 4.653	0.921	20.01 ± 3.219	0.071	16.69 ± 6.038	0.000*
	Female	4.74 ± 1.155		16.56 ± 4.230		20.54 ± 3.254		19.45 ± 5.531	
	Prefer not to say	4.83 ± 1.329		17.67 ± 2.338		20.00 ± 4.000		18.67 ± 6.055	
Area	Rural	4.53 ± 1.138	0.026*	15.93 ± 4.363	0.027*	20.01 ± 3.887	0.002*	17.15 ± 6.841	0.009*
	Urban	4.81 ± 1.116		16.70 ± 4.322		20.49 ± 3.041		19.08 ± 5.409	
Education	None	4.50 ± 1.291	0.125	17.75 ± 4.717	0.062	22.00 ± 2.828	0.041*	21.25 ± 8.261	0.032*
	No certificate but can read/write	4.67 ± 1.528		11.00 ± 9.644		20.67 ± 1.528		15.00 ± 10.817	
	Primary	-		-		-		-	
	Matriculation	4.07 ± 0.458		16.53 ± 3.182		21.13 ± 3.796		19.20 ± 7.143	
	Intermediate	4.83 ± 1.186		16.02 ± 4.448		19.76 ± 3.544		18.46 ± 5.945	
	University graduate	4.75 ± 1.153		16.51 ± 4.290		20.33 ± 3.230		18.37 ± 5.671	
	Above	4.79 ± 0.952		17.39 ± 4.268		21.15 ± 2.811		20.17 ± 5.674	
Marital status	Married	4.71 ± 0.984	0.667	16.95 ± 3.797	0.036*	20.28 ± 3.268	0.775	18.56 ± 5.463	0.818
	Single	4.76 ± 1.155		16.43 ± 4.446		20.40 ± 3.254		18.66 ± 5.891	
Employment	Employed	4.70 ± 1.101	0.394	16.73 ± 4.310	0.282	20.52 ± 2.959	0.804	18.07 ± 5.906	0.098
	Unemployed	4.77 ± 1.137		16.44 ± 4.355		20.32 ± 3.376		18.90 ± 5.761	

* P–value < 0.05 was considered statistically significant, Mann-Whitney U test, and Kruskal-Wallis test were used when Shapiro-Wilk test gave significant results.

**Table 3.** Regression analysis for the determination of the influence of knowledge score on beliefs, practices, and adherence

	Covariates	Knowledge	Beliefs	Practices	Adherence
**Age**	**B**	0.055	0.045	−0.045	0.031
	**Lower bound**	−0.075	−0.346	−0.691	−0.571
	**Upper bound**	0.257	0.920	0.269	1.094
	***P*** **value**	0.282	0.374	0.387	0.538
**Gender**	**B**	−0.028	0.049	0.084	0.211
	**Lower bound**	−0.273	−0.337	−0.021	1.573
	**Upper bound**	0.141	1.244	1.178	3.653
	***P*** **value**	0.531	0.261	0.058	0.000*
**Area**	**B**	0.108	0.048	0.048	0.115
	**Lower bound**	0.076	−0.324	−0.254	0.514
	**Upper bound**	0.504	1.322	0.994	2.680
	***P*** **value**	0.008*	0.234	0.244	0.004*
**Education**	**B**	0.025	0.055	0.049	0.041
	**Lower bound**	−0.083	−0.153	−0.142	−0.296
	**Upper bound**	0.155	0.758	0.549	0.903
	***P*** **value**	0.552	0.192	0.247	0.320
**Marital status**	**B**	0.038	−0.015	0.000	−0.004
	**Lower bound**	−0.160	−1.207	−0.791	−1.435
	**Upper bound**	0.386	0.879	0.790	1.309
	***P*** **value**	0.418	0.757	0.999	0.928
**Employment**	**B**	0.063	−0.022	−0.067	0.010
	**Lower bound**	−0.066	−1.049	−1.109	−0.983
	**Upper bound**	0.373	0.632	0.166	1.228
	***P*** **value**	0.171	0.626	0.147	0.828

Multiple logistic regression was applied, section scores were considered as the dependent variable, * *P* –value < 0.05 was considered statistically significant, β = standardized beta.

#### Adherence to hand hygienic guidelines

Overall, participants showed poor adherence to hygienic guidelines. There was a statistically significant gender-based (*P* < 0.01) and area-based (*P* = 0.009) differences in the scores. Regression model revealed that females were more adherent (β = 0.211, CI = 1.573 − 3.653, *P* < 0.01) to the guidelines as compared to males. There was a significant association (β = 0.102, CI = 0.119 − 0.932, *P* = 0.011) between participants’ knowledge and adherence score. 64.2% (395) of the people disinfected their frequently used items, 84.7% (522) coughed or sneezed into tissue or the inside of elbow, 87.8% (541) wore facemask in public and 53% (327) avoided touching their face unnecessarily.

#### Barriers to optimal hand hygienic practices

**Figure 2.**
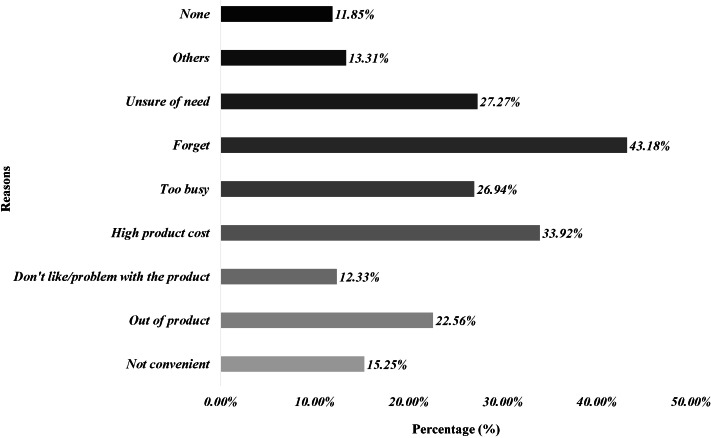
Factors affecting compliance with optimal hand hygiene practices during coronavirus pandemic.

When asked about the barriers towards optimal hand hygiene, forgetfulness (43.2%), high product cost (33.9%), unsure of need (27.3%), and busy life (26.9%) were the most chosen options by the respondents. Male participants had most of the reasons not to comply with the optimal hand hygienic practices, while females were mostly unbothered by most of the barriers. Figure 2 highlights different barriers, in view of the participants, that may interfere with their compliance to optimal hand hygienic practices.

## Discussion

Good hand hygienic practices are considered an important factor to curb the transmission and emergence of SARS-CoV-2.^18^ Our investigative survey demonstrates that most individuals reported poor hand hygienic practices during the peak pandemic period. Similar findings were reported in previous study conducted at the beginning of H1N1 influenza pandemic.^19^ Overall, respondents had average knowledge about the hand hygienic practices, but there was a significant difference (*P* = 0.026) in the knowledge scores of rural and urban residents regardless of their education level. This could be due to misperception about the perceived benefits of adopting good hygienic practices as reported in previous studies.^20^ Most of the respondents believed in the effectiveness and perceived outcomes of good hygienic practices; however, there was significant difference (*P* = 0.036) among different age groups about behavioral beliefs regarding hand hygienic practices. Results from linear regression revealed that behavioral beliefs were significantly associated (β = 0.157, CI = 0.302 − 0.905, *P* < 0.01) with the knowledge score. Similar findings were reported in a study conducted earlier during SARS-CoV-2 pandemic.^21^

Most participants believed that they were protecting their own health and the health of their family by performing good hygienic practices. The difference in age groups could be reduced by educating the individuals, which in turn would enhance the knowledge regarding infectious diseases. Hygiene education should be inducted, and stakeholders must raise the awareness about the importance of performing good hand hygienic practices. Hand hygienic practices of the people did not improve during the peak of the pandemic in Pakistan, and there was a significant difference (*P* = 0.041) in hand hygienic practices among people of different educational backgrounds. This shows the importance of educating the public in order to curb the transmission of SARS-CoV-2. Stakeholders in conjunction with the healthcare professionals can play an important role in the dissemination of information and educating communities before this virus could wreak more havoc.^22^ It is worth mentioning that the higher scores of knowledge were significantly associated (β = 0.043, CI = 0.105 − 0.353, *P* = 0.003) with lower likelihood of potentially poor hand hygienic practices. Hand hygienic campaigns through mass media must be initiated, that are modelled on World Health Organization’s multimodal hand hygienic guidelines and strategies.^23^

Although many respondents prioritized hand washing whenever they came home from outside, and washed their hands for at least 20 seconds, there was a significant difference in the practices of rural and urban population. There is a difference in culture, beliefs, and norms between the rural and urban areas of Pakistan at so many levels, that most of the rural people didn’t even believe in the existence of coronavirus pandemic at first.^24^ This denial phase was somewhat reduced by educating the public and increasing awareness regarding hand hygienic practices.^25^ However, a lot of work still has to be done in narrowing the knowledge and practice gap between the rural and urban wings of Pakistan.

Most of the participants were non-adherent to the healthcare guidelines provided by stakeholders, and there was a significant difference among some groups.^26,27^ Very few participants avoided social gatherings and tried to minimize outside exposure as much as they could by working at home, drinking or eating at home and avoiding social visits other than buying essential groceries from the market, etc. However, many participants wore face masks in public places, disinfected their hands every now and then, and maintained social distance, but frequencies of such activities varied among different people.

Adherence to the guidelines could be enhanced by increasing public knowledge, as knowledge score had significant association (β = 0.102, *P* = 0.011) with adherence. Although, the Government of Pakistan released time to time guidelines for general public and healthcare professionals to effectively curb the emergence of coronavirus pandemic,^26,27^ its implementation remained quite controversial throughout the pandemic.^28^ Number of cases rose due to the ineffectiveness of stakeholders to implement guidelines and educate the masses through proper channels.^29^ This difference in adherence to the guidelines can be reduced by strict implementation of government and stakeholder’s policies. Similar findings can also be seen in another study conducted in mainland China during the coronavirus pandemic.^30^

If frequent hand hygienic practices are ignored, then a second wave of coronavirus pandemic is inevitable. Previous experiences have taught us that people usually get tired of doing the same thing and listening to the same news every day.^30^ Ultimately, they become even more careless, which increases the risk of a very painful second wave. That is an important lesson we should learn if we want to control this pandemic from happening again. COVID-19 pandemic has taught us to respond smartly and quickly, with the right approach of mass tracing and testing rather than relying on the lockdowns only. The results of our activities in the first wave will determine the duration and magnitude of the second wave; therefore, we should focus more on good hand hygienic practices along with other precautionary measure to prevent the emergence of second wave and minimize the future need of lockdowns.

## Limitations

Although this study recruited more than the recommended sample size, it is still not enough to generalize the outcomes of this study in any country other than Pakistan. Compared to other studies, our sample was an overestimate of females and educated people. So, there may be a chance of selection bias and the outcomes of this study may only be generalized to the people of high socio-economic status. Due to limited access of internet and online resources in certain areas of Pakistan, this study couldn’t get to such grassroots areas. We can only expect that people of such areas to have poor knowledge and practices regarding hand hygiene. Moreover, this was a self-reported questionnaire-based study and no psychological techniques were applied to assess the veracity of responses, so there may be a chance of reporting bias.

## Conclusion

Overall, the hand hygienic practices of the people were poor and there was a significant difference with respect to educational status. Most of the participants were non-adherent to hygienic guidelines. There was a significant association between public adherence and knowledge regarding hand hygienic practices. Many people were unsure of the need of adopting optimal hand hygienic practices and considered it, among many other factors, as a potential barrier to their compliance with good hand hygienic practices. This highlights the need for interventions by stakeholders and instigation of a robust campaign that focuses on educating the public through targeted approach. Pictograms that describe the method of hand washing and urging the public to take prompt action in this regard, could be spread through electronic media, newspapers, billboards, etc. Special commercials and advertisements on television with appealing slogans that enhance consciousness to this global issue, could be effective in making people more compliant to optimal hand hygienic practices.
